# Is There an Effect of Initial and 24-Hour Blood Gas Lactate and Methemoglobin Levels on Predicting Mortality of Patients in the Intensive Care Unit?

**DOI:** 10.3390/life15030373

**Published:** 2025-02-26

**Authors:** Serhat Doğan, Sefer Aslan, Tayfun Börta, Mehmet Sarıaydın, Hakan Sezgin Sayıner

**Affiliations:** 1 Private Kayseri Acıbadem Hospital, General Surgery, Kayseri 38140, Turkey; 2 Giresun University Medicine School, Internal Medicine, Giresun 28200, Turkey; drseferaslan02@hotmail.com; 3 Adıyaman Education and Research Hospital, Internal Medicine, Adıyaman 02100, Turkey; tayfunborta@hotmail.com; 4 Medical Park İstanbul Hospital, Internal Medicine, İstanbul 34899, Turkey; sariaydinmehmet@gmail.com; 5 Adıyaman University Medicine School, Infectious Disease, Adıyaman 02200, Turkey; drhssayiner@yahoo.com

**Keywords:** lactate, blood gas, methemoglobine, mortality, ICU

## Abstract

In intensive care units (ICUs), serum lactate and methemoglobin (metHb) levels are considered significant biomarkers for predicting mortality in critically ill patients. This study investigates the relationship between lactate and metHb levels in blood gas analyses at admission and 24 h later, as well as their association with mortality in ICU patients. The study was conducted retrospectively between March and December 2022 at Adıyaman Training and Research Hospital, evaluating 114 patients, with statistical analyses performed on the collected data. The results indicated a statistically significant decrease in lactate levels between admission and 24 h after (*p* = 0.004). However, no significant change was found in metHb levels (*p* > 0.05). Lactate clearance was significantly lower in deceased patients compared to survivors (*p* = 0.037), whereas metHb clearance showed no statistically significant association with mortality. Lactate is highlighted as a key indicator of tissue hypoxia and plays a critical role in managing critically ill patients. Elevated lactate levels are associated with impaired oxygenation and worse prognoses. The literature consistently supports the association between high lactate levels and increased mortality in conditions such as sepsis and hemorrhagic shock. Similarly, this study confirms the prognostic value of lactate, particularly in the early phases of ICU admission. In contrast, metHb levels were not found to significantly impact mortality. Although some studies suggest a potential role of metHb as a biomarker for oxidative stress in inflammatory diseases, this relationship was not supported by the current findings. In conclusion, serum lactate levels serve as a crucial tool for mortality prediction and patient management in ICUs, while metHb levels have limited prognostic value. These findings suggest that greater emphasis should be placed on lactate monitoring in the management of critically ill patients.

## 1. Introduction

The relationship between life and death has long captivated human inquiry, driving advancements in medical science through the exploration of mortality mechanisms and the development of predictive tools. In modern medicine, accurately forecasting mortality in critically ill patients admitted to intensive care units (ICUs) is essential for optimizing patient outcomes. Such predictions allow clinicians to tailor therapeutic interventions, promptly identify deficiencies, and establish individualized care plans. Among the various factors influencing mortality, tissue hypoxia is particularly critical, with blood lactate levels serving as a key indicator of its presence.

Advances in medical technology have made the measurement of blood lactate levels more accessible, rapid, and cost-effective. Lactate can be quantified from arterial, venous, or capillary blood samples, offering clinicians a versatile tool for assessment. Numerous studies have established a strong correlation between serum lactate levels and patient prognosis in critical care settings. Elevated lactate levels often indicate organ dysfunction and tissue hypoperfusion, and they have proven valuable as early markers of clinical severity, aiding in the prediction of outcomes and in reducing mortality rates in ICU patients [1,2,3].

However, a single lactate measurement may not capture the dynamic metabolic and pathological changes occurring in critically ill patients. Emerging evidence suggests that lactate clearance—the rate at which lactate levels decrease—may serve as a more robust prognostic marker than absolute lactate values. By evaluating lactate clearance, clinicians can gain deeper insights into capillary perfusion, disease progression, and the effectiveness of therapeutic interventions [4,5].

Methemoglobinemia represents another critical factor in ICU management. It arises from the oxidation of the ferrous ion (Fe^2^⁺) in oxyhemoglobin to the ferric ion (Fe^3^⁺), leading to impaired oxygen delivery to tissues. Clinical manifestations of methemoglobinemia vary according to methemoglobin levels, ranging from tachycardia and dyspnea at moderate levels to confusion, loss of consciousness, or even fatal outcomes when levels exceed 50% [6,7,8]. Although historical studies have detailed the clinical features and risks associated with methemoglobinemia, recent research has begun to elucidate its potential role as a marker of oxidative stress and inflammation in critically ill patients. Some recent investigations suggest that elevated methemoglobin levels may be linked to adverse outcomes in sepsis and other critical illnesses, highlighting the need for further validation and comprehensive review of its prognostic value [9,10].

### Hypothesis and Objectives

Based on the above considerations, we hypothesize that dynamic changes in blood gas lactate and methemoglobin levels during the first 24 h of ICU admission are predictive of in-hospital mortality in critically ill patients. Specifically, this study aims to:

1. Evaluate the prognostic significance of initial lactate levels and 24-h lactate clearance in predicting mortality among ICU patients.
2. Assess the association between methemoglobin levels and clinical outcomes, incorporating recent findings on its role as a marker of oxidative stress and inflammation.
3. Compare the predictive performance of lactate and methemoglobin measurements in forecasting mortality, thereby determining their relative utility in guiding clinical decision-making in the ICU.

This study seeks to provide enhanced insights into the dynamic behavior of these biomarkers and their potential roles in early risk stratification and management of critically ill patients.

## 2. Methods

### 2.1. Study Design and Setting

This retrospective observational study was conducted in the Internal Medicine Intensive Care Unit (ICU) of Adıyaman Training and Research Hospital between 15 March 2022 and 1 December 2022. Adıyaman University Ethics committee approval dated 19 November 2024 and numbered 2024/9-15 was obtained for the non-interventional study.

The primary aim was to investigate the prognostic significance of arterial blood gas lactate and methemoglobin (metHb) levels measured at ICU admission and again at the 24th hour in predicting patient mortality. The final sample size of 114 patients was determined by the availability of complete data during the study period; a post-hoc power analysis confirmed that the study had over 80% power to detect significant differences between survivors and non-survivors.

### 2.2. Patient Selection and Inclusion Criteria

The study population included adult patients (aged ≥ 18 years) admitted to the ICU who had arterial blood gas analyses performed both at the time of admission and at the 24th hour of their ICU stay. Patients were included regardless of their underlying diagnosis or the primary reason for ICU admission.

Exclusion criteria were applied to minimize confounding and ensure data completeness, and included:

- Missing or incomplete blood gas analysis data.
- Unavailability of lactate or metHb measurements at either time point.
- Discharge or transfer before the 24-h measurement could be obtained.
- Presence of chronic hematological disorders, such as chronic hemolytic anemia, or congenital methemoglobinemia, which could affect baseline metHb levels.
- Use of medications known to induce methemoglobinemia.

### 2.3. Data Collection and Variables

Patient data were retrieved from the hospital’s electronic medical records and included demographic information (age, gender), clinical variables (comorbid conditions, severity of illness scores when available, and ICU length of stay), and mortality status.

The primary variables of interest were the arterial blood gas lactate and metHb levels measured at ICU admission and at the 24th hour. Blood samples were collected using standardized protocols and analyzed in the hospital’s central laboratory with a consistent methodology across all patients. Lactate levels were expressed in mmol/L, with a predefined cutoff value of 0.9 mmol/L based on previous studies demonstrating high sensitivity and specificity in predicting mortality. The metHb levels were recorded concurrently and evaluated for their potential role as prognostic biomarkers. The primary endpoint of the study was overall ICU mortality, defined as death occurring during the ICU stay.

### 2.4. Statistical Analysis

Data analysis was performed using IBM SPSS Statistics version 22. The normality of continuous variables was assessed using the Kolmogorov–Smirnov test. Descriptive statistics were presented as the mean ± standard deviation or median (interquartile range) for continuous variables and as frequency distributions for categorical variables.

For comparisons between survivors and non-survivors, non-normally distributed continuous variables were analyzed using the Mann–Whitney U test, and categorical variables were compared using the chi-square test or Fisher’s exact test, as appropriate. The Wilcoxon signed-rank test was employed to evaluate changes in lactate and metHb levels over time. Spearman’s rho correlation coefficient was used to assess relationships between continuous variables.

Receiver operating characteristic (ROC) curve analysis was conducted to determine the optimal cutoff values for lactate and metHb levels in predicting ICU mortality; sensitivity, specificity, and area under the curve (AUC) values were calculated to assess the diagnostic performance of these biomarkers. In addition, multivariate logistic regression analysis was performed to adjust for potential confounders—such as age, comorbidities, and severity of illness scores—to determine the independent prognostic value of lactate and metHb levels. A significance level of *p* < 0.05 was considered statistically significant.

By employing a comprehensive statistical approach and addressing potential confounding factors, this study aimed to elucidate the associations between dynamic changes in lactate and metHb levels and ICU mortality, thereby providing valuable insights into their prognostic utility in critically ill patients.

## 3. Results

The study included a total of 114 ICU patients, with ages ranging from 21 to 103 years (mean age: 69.62 ± 17.62 years). Of these, 52 patients (45.6%) were female, while 62 (54.4%) were male. At the time of data evaluation, 72 patients (63.2%) had survived, whereas 42 patients (36.8%) had succumbed to their illnesses during their ICU stay.

The most frequently observed comorbid conditions among the study population included acute and chronic kidney failure (32.1%), cerebrovascular thrombosis (26.8%), and pneumonia (24.1%). Other notable conditions included malignancy (16.1%), congestive heart failure (12.5%), and gastrointestinal bleeding (7.1%). A detailed distribution of comorbidities is provided in Table 1.

A statistically significant decrease in lactate levels was observed between ICU admission and the 24th hour of hospitalization (*p* = 0.004). The mean lactate level at admission was 3.71 ± 2.86 mmol/L (median: 3.0), which declined to 2.96 ± 1.31 mmol/L (median: 2.8) at the 24 h mark.

In contrast, the changes in metHb levels between the two time points were not statistically significant (*p* = 0.211). The mean metHb level at admission was 2.10 ± 1.06% (median: 2.4), and at the 24th hour, it was recorded as 1.96 ± 0.97% (median: 2.3). These findings suggest that while lactate levels exhibit a notable reduction over time, metHb levels remain relatively stable (Table 2).

Lactate clearance was found to be significantly lower in deceased patients compared to those who survived (*p* = 0.037). The mean lactate clearance for survivors was 1.53 ± 1.13 mmol/L (median: 1.22), whereas for deceased patients, it was lower, at 1.16 ± 0.70 mmol/L (median: 1.05). These findings highlight the potential role of lactate clearance as a prognostic indicator in critically ill patients.

**Figure 1:**
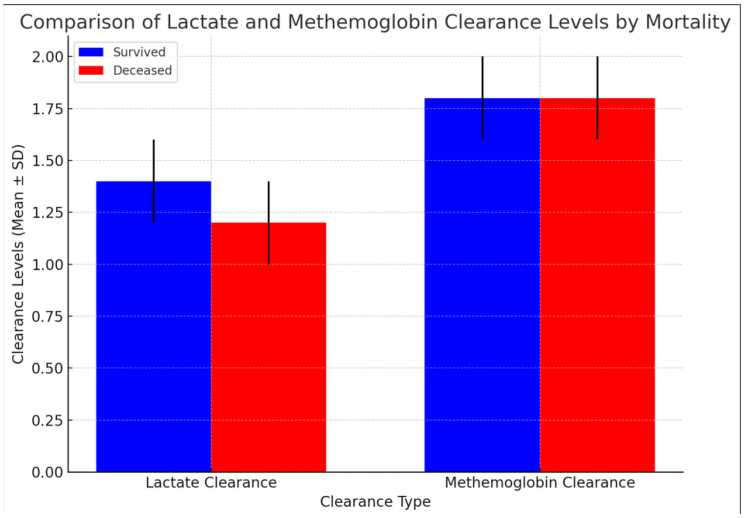
Graph of lactate clearance and methemoglobin clearance levels based on mortality.

On the other hand, metHb clearance did not demonstrate a statistically significant difference between survivors and deceased patients (*p* = 0.315). The mean metHb clearance was 1.74 ± 2.29% (median: 1.04) in the survivor group and 1.74 ± 2.11% (median: 1.00) in the deceased group, suggesting that metHb clearance lacks predictive utility in this context (Table 3) (Figure 1).

No statistically significant difference was observed in ICU length of stay between survivors and deceased patients (*p* = 0.151). The mean ICU stay was 7.28 ± 7.21 days (median: 5 days) for survivors and 8.38 ± 7.01 days (median: 6 days) for deceased patients, indicating that length of hospitalization did not appear to be a determining factor in patient mortality (*p* = 0.151) (Table 4).

Furthermore, no significant correlation was found between ICU length of stay and either lactate clearance (*p* = 0.177) or metHb clearance (*p* = 0.805), suggesting that these parameters do not influence the duration of ICU hospitalization (Table 5).

**Figure 2:**
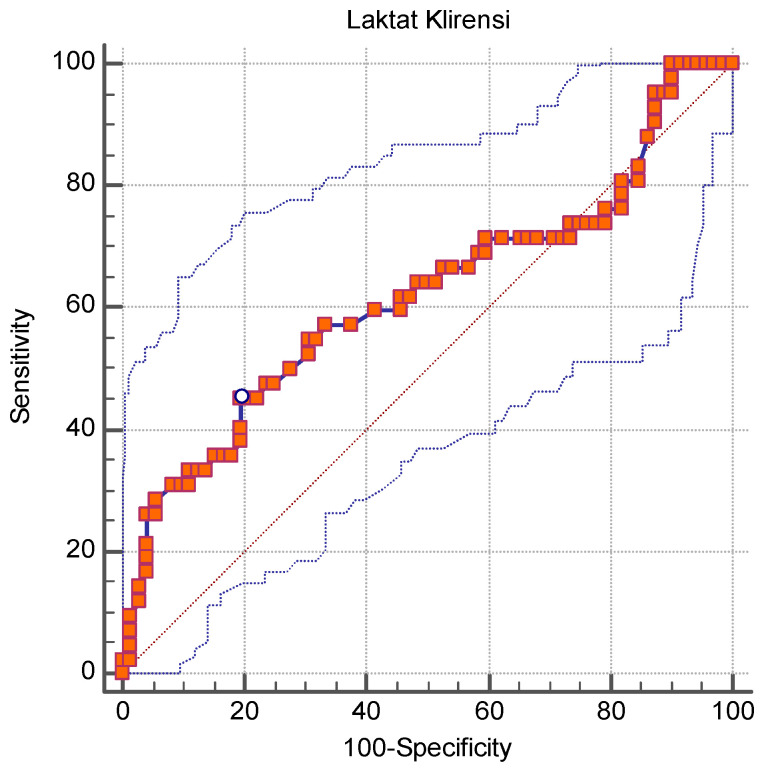
ROC curve for lactate clearance in predicting mortality.

Receiver operating characteristic (ROC) curve analysis for lactate clearance yielded an area under the curve (AUC) of 0.618 (95% CI: 0.518–0.718, *p* = 0.043), indicating a moderate ability to predict ICU mortality. The optimal cutoff value for lactate clearance was determined as ≤0.9 mmol/L, with a sensitivity of 45.2% and a specificity of 80.6% (Figure 2).

The moderate AUC underscores the limited discriminative power of lactate clearance despite its statistically significant association with mortality.

Given the relatively small sample size (*n* = 114), sensitivity analyses were performed by excluding patients with missing comorbidity data or extreme biomarker values. These analyses did not materially change the observed associations between lactate clearance and mortality, thereby supporting the robustness of our findings.

Readings at 24 h post-admission did not demonstrate significant differences between survivors and deceased patients. At ICU admission, the mean lactate/metHb ratio was 3.66 ± 6.44 (median: 1.6) for survivors and 3.29 ± 4.97 (median: 1.2) for deceased patients (*p* = 0.368). Similarly, at the 24 h mark, the mean lactate/metHb ratio was 2.83 ± 4.77 (median: 1.36) for survivors and 3.64 ± 8.79 (median: 1.39) for deceased patients (*p* = 0.891). These findings suggest that the lactate/metHb ratio does not serve as a reliable predictor of mortality in critically ill patients (Table 6).

## 4. Discussion

The results of this study highlight the prognostic significance of lactate clearance in predicting mortality among ICU patients, whereas methemoglobin levels and clearance appear to have limited predictive utility. The significant decrease in lactate levels over time, coupled with its lower clearance in deceased patients, suggests that monitoring lactate dynamics may aid in risk stratification and early identification of critically ill patients at higher risk of mortality. In contrast, metHb levels did not exhibit significant changes over time or associations with mortality, indicating that their role in ICU prognosis remains uncertain.

In summary, our results highlight that lactate clearance is significantly associated with ICU mortality, suggesting its potential role as a prognostic indicator. In contrast, metHb levels and their clearance, as well as the lactate/metHb ratio, appear to have limited predictive utility in this patient population.

Additionally, ICU length of stay was not significantly correlated with mortality or lactate/metHb clearance, suggesting that other clinical factors may play a more substantial role in determining patient outcomes.

Overall, these findings reinforce the importance of lactate clearance as a valuable biomarker in ICU patient management, while metHb levels appear to be less relevant in mortality prediction. Further studies with larger patient populations and prospective study designs may be required to validate these results and explore potential clinical applications.

Predicting mortality in critically ill patients is closely related to tissue oxygenation. The lactate level is a key parameter for determining tissue hypoxia. Methemoglobin (metHb) levels increase as a result of oxidative stress, and they have been investigated as potential biomarkers for the early diagnosis and long-term prognosis of various inflammatory diseases.

Schuerholz et al. retrospectively evaluated metHb levels in 655 critically ill patients and found that septic patients had significantly higher metHb levels at the time of admission to intensive care units compared to non-septic patients. They also observed a significant correlation between these increased levels and changes in Sequential Organ Failure Assessment (SOFA) scores [9].

Similarly, Ohashi et al. reported that septic patients had higher metHb levels than non-septic patients in the same intensive care unit. However, in our study, metHb levels did not show a statistically significant relationship with mortality between septic and non-septic patients (*p* > 0.05). Ohashi and colleagues evaluated metHb through serial measurements during the recovery phase of sepsis [10]. In our study, when comparing metHb levels obtained from blood gas analysis at admission and at 24 h, no significant difference related to mortality was observed.

Consistent with the literature, our findings demonstrate that metHb is not a useful prognostic marker for mortality in intensive care patients (Table 3). It is well established that lactate levels increase rapidly due to various causes of tissue hypoxia [11].

Singer et al. conducted serial lactate measurements in 258 sepsis patients monitored in the emergency department and found that elevated lactate levels were clearly associated with poor prognosis [12]. Our study included fewer patients than Singer et al.’s but encompassed all patients in the intensive care unit, focusing on lactate levels from arterial blood gas analyses at admission and 24 h after (Table 2). Thus, our study supports Singer et al.’s findings with a broader diagnostic scope and fewer laboratory analyses.

Kruse et al. systematically reviewed the predictive value of admission lactate levels for mortality and emphasized that elevated lactate levels at admission were associated with increased mortality. They reported that patients with lactate levels above 2.5 mmol/L had significantly higher mortality rates [13]. Similarly, our study showed that elevated lactate levels in deceased patients were significantly associated with mortality (*p* < 0.05). Lactate levels were notably higher in deceased patients than in survivors. Consequently, lactate levels at admission may provide critical information to clinicians regarding mortality, even at the initial assessment (Table 2 and Table 3).

Smith et al. conducted a prospective study on 148 intensive care unit (ICU) patients with no specific disease categorization. They analyzed lactate levels measured in blood gases at the time of hospital admission and in arterial blood gases at the 24th hour. A cut-off value of 1.5 mmol was accepted for lactate. It was found that patients with lactate levels higher than 1.5 mmol upon ICU admission had higher hospital mortality. Additionally, if the blood lactate level measured after 24 h was higher than 1.0 mmol, the mortality rate was significantly increased (*p* = 0.0001) [14].

Van Beest et al. performed a prospective study on 135 patients aged 18 years or older who exhibited shock symptoms for at least 2 h. In samples taken from patients’ capillary and venous blood, a strong correlation was found between hyperlactatemia and mortality. A lactate cut-off value of 4.0 mmol was accepted [15].

Arnold et al. conducted a retrospective study involving 166 sepsis patients aged 17 years and older who were presented to the emergency department. Sequential venous blood lactate sampling was performed on these patients. The lactate cut-off value was set at 4.0 mmol. In this study, it was observed that mortality increased when the initial lactate level of deceased patients exceeded 4.7 mmol. This study also demonstrated that appropriate treatment could reduce blood lactate levels in patients with septic or circulatory shock [16].

Jansen et al. conducted a prospective study on 394 ICU patients treated for sepsis, hemorrhagic shock, or other conditions causing reduced oxygen transport. Arterial lactate sampling was performed at the 12th and 24th hours. They observed that a reduction in lactate levels at the 24th hour in septic patients was associated with decreased mortality. In this study, a lactate cut-off value of 2.0 mmol was used [17].

In contrast, methemoglobin (metHb) levels, which can rise in response to oxidative stress, have been investigated as potential biomarkers for the diagnosis and prognosis of inflammatory and oxidative stress-related conditions. Prior research by Schuerholz et al. and Ohashi et al. reported that septic patients tended to have higher metHb levels at ICU admission and that these levels correlated with disease severity indicators such as the Sequential Organ Failure Assessment (SOFA) score. However, in our study, metHb levels obtained from arterial blood gas analyses at both admission and 24 h after did not exhibit a statistically significant relationship with mortality (*p* > 0.05). This discrepancy may be due to several factors, including the timing of sample collection, the transient nature of metHb elevations, or differences in the patient population. While our findings suggest that metHb is not a useful prognostic marker in the overall ICU cohort, it remains possible that metHb could have prognostic value in more narrowly defined subgroups of patients, particularly those in whom oxidative stress plays a more dominant role. Therefore, rather than dismissing metHb entirely, our study highlights the need for further investigation into its potential utility when combined with other markers or assessed under different clinical conditions.

A balanced interpretation of our results must consider the limitations inherent in our study design. Our study was retrospective in nature, which introduces selection bias and limits our ability to establish causal relationships between the measured biomarkers and patient outcomes. Moreover, as this was a single-center study, the findings may be influenced by local patient demographics, treatment protocols, and laboratory practices, potentially limiting the generalizability of the results. Missing data and unmeasured confounders—such as variations in comorbid conditions, therapeutic interventions, and severity of illness scores—might have also affected our analyses. Although we attempted to adjust for known confounders in our statistical models, residual confounding cannot be completely ruled out.

The retrospective design also means that our study relied on available clinical data and blood gas analyses performed at only two time points (at admission and after 24 h). This approach, while practical, may not capture the full dynamic profile of lactate and metHb levels throughout the entire course of a patient’s ICU stay. Prospective studies with more frequent sampling intervals could provide deeper insights into the temporal evolution of these biomarkers and their relationship with patient outcomes.

Given these limitations and the relatively modest sample size of our study, future research should focus on prospective, multicenter studies to validate our findings. Such studies should aim to:

- Standardize the measurement protocols for lactate and metHb levels to reduce inter-laboratory variability.
- Expand the sample size and diversity of the patient population to enhance the generalizability of the results.
- Include serial measurements over a longer duration to better understand the kinetics of these biomarkers.
- Explore the combination of lactate and metHb with other biomarkers (such as inflammatory cytokines or markers of organ dysfunction) to develop a more comprehensive prognostic model.

This multifaceted approach would not only confirm the utility of lactate as a prognostic indicator but also help clarify the potential role of metHb as part of a composite biomarker panel for predicting mortality and guiding therapeutic interventions in critically ill patients.

## 5. Conclusions

While the prognostic role of lactate in critical care is well-established, this study reinforces its clinical utility by emphasizing the dynamic assessment of lactate clearance as a pivotal marker for mortality risk stratification. The inclusion of methemoglobin (metHb) introduces a novel dimension, addressing a gap in the existing literature, as prior studies have predominantly focused on static lactate measurements or overlooked oxidative stress biomarkers. Although our findings did not substantiate metHb as an independent predictor of mortality, the direct comparison of lactate and metHb dynamics over time provides fresh insights into their divergent behaviors in critically ill patients. This temporal analysis underscores the importance of evaluating biomarker trajectories rather than isolated values. Future research should explore metHb in specific subpopulations—such as those with pronounced oxidative stress (e.g., sepsis or drug-induced toxicity)—or investigate its synergistic value when combined with inflammatory or organ dysfunction markers. Such approaches may unveil context-dependent prognostic roles for metHb, refining risk prediction models in critical care.

### 5.1. Limitations

Retrospective Design: This study’s retrospective nature may introduce selection bias and limit the ability to establish causation between serum markers and mortality.

Single-Center Study: Data were collected from a single institution, which may reduce the generalizability of the findings to other settings or populations.

Sample Size: The relatively small sample size of 114 patients may limit the statistical power and the ability to detect subtle associations, particularly for methemoglobin levels.

Lack of Comprehensive Clinical Data: Other potential confounding factors, such as comorbidities, treatments, or interventions, were not extensively analyzed and may influence the observed associations.

Focus on Early ICU Period: The study only analyzed blood gas results at admission and after 24 h, which may not capture longer-term trends or dynamics in lactate and methemoglobin levels.

Limited Investigation of metHb Utility: The role of methemoglobin in specific inflammatory or oxidative conditions was not thoroughly explored, potentially underestimating its relevance in certain subgroups of ICU patients.

#### Implications and Recommendations for Practice

Routine Lactate Monitoring: The findings underscore the importance of incorporating regular lactate level assessments in the management of ICU patients. Elevated lactate levels and poor lactate clearance should prompt clinicians to evaluate for tissue hypoxia and implement timely interventions.

Early Risk Stratification: Lactate levels can serve as a reliable marker for early risk stratification in critically ill patients, enabling healthcare providers to prioritize care for high-risk individuals and potentially improve outcomes.

Focus on Proven Markers: While methemoglobin levels were not predictive of mortality in this study, the emphasis should remain on validated markers like lactate for guiding clinical decision-making in ICU settings.

Continuous Education and Protocol Development: Clinicians should be trained on the prognostic significance of lactate clearance and its integration into ICU protocols to enhance patient care strategies.

Future Research Directions: Further studies should explore larger and more diverse patient populations to validate these findings and investigate the potential utility of methemoglobin levels in specific clinical scenarios.

### 5.2. Relevance to Clinical Practice

This study emphasizes the critical role of lactate monitoring in ICU settings, highlighting its value as a prognostic marker for mortality risk and its utility in guiding patient management. Routine assessment of lactate levels can aid in the early identification of high-risk patients, enabling timely and targeted interventions. Conversely, the limited prognostic relevance of methemoglobin levels suggests that clinical focus should remain on more robust markers like lactate for optimizing outcomes in critically ill patients.

## Figures and Tables

**Table 1:** Distribution of comorbidities (n = 112).

Comorbidities	*n*	%
Gastrointestinal bleeding	8	7.1
Thrombosis (CVD)	30	26.8
Hemorrhagic (CVD)	4	3.6
Diabetic Ketoacidosis (DKA)	5	4.5
Malignancy	18	16.1
Pneumonia	27	24.1
Congestive heart failure (CHF)	14	12.5
Pulmonary embolism	1	0.9
Intoxication	1	0.9
Sepsis	1	0.9
Acute and chronic kidney failure	36	32.1

Note: Results for two patients were excluded due to missing data. CVD: Cerebrovascular disease.

**Table 2:** Changes in lactate and methemoglobin levels between admission and 24 h after.

Variable	Mean ± SD (Median)	Mean ± SD (Median)	*p*-Value
	Admission	24 h	
Lactate (mmol/L)	3.71 ± 2.86 (3)	2.96 ± 1.31 (2.8)	0.004 *
Methemoglobin (%)	2.10 ± 1.06 (2.4)	1.96 ± 0.97 (2.3)	0.211

* Wilcoxon signed-rank test, *p* < 0.05 is considered statistically significant.

**Table 3:** Evaluation of lactate and methemoglobin clearance based on mortality.

Clearance	Mean ± SD (Median)	Mean ± SD (Median)	*p*-Value
	Survived	Deceased	
Lactate Clearance	1.53 ± 1.13 (1.22)	1.16 ± 0.70 (1.05)	0.037 *
Methemoglobin Clearance	1.74 ± 2.29 (1.04)	1.74 ± 2.11 (1)	0.315

* Mann–Whitney U test, *p* < 0.05 is considered statistically significant.

**Table 4:** Evaluation of hospital stay based on mortality.

Variable	Mean ± SD (Median)	Mean ± SD (Median)	*p*-Value
	Survived	Deceased	
Length of Stay (days)	7.28 ± 7.21 (5)	8.38 ± 7.01 (6)	0.151

Mann–Whitney U test.

**Table 5:** Correlation between length of stay and clearance levels.

Variable	r	*p*-Value
Lactate Clearance	−0.127	0.177
Methemoglobin Clearance	0.025	0.805

Spearman’s rho correlation test.

**Table 6:** Evaluation of lactate/methemoglobin ratios based on mortality.

Variable	Mean ± SD (Median)	Mean ± SD (Median)	*p*-Value
	Survived	Deceased	
Lactate/Methemoglobin (Admission)	3.66 ± 6.44 (1.6)	3.29 ± 4.97 (1.2)	0.368
Lactate/Methemoglobin (24 h)	2.83 ± 4.77 (1.36)	3.64 ± 8.79 (1.39)	0.891

Mann–Whitney U test.

## Data Availability

The data presented in this study are available on request from the corresponding author due to legal limitations.
